# Current Trends in Diagnosis, Treatment and Prognosis of Canine Insulinoma

**DOI:** 10.3390/vetsci9100540

**Published:** 2022-09-29

**Authors:** Floryne O. Buishand

**Affiliations:** Department of Clinical Sciences and Services, Royal Veterinary College, Hertfordshire AL9 7TA, UK; fbuishand@rvc.ac.uk

**Keywords:** pancreatic neuroendocrine tumours, dog, insulinomas, pancreas

## Abstract

**Simple Summary:**

Dogs can develop a tumour of the pancreas that produces too much insulin, which is called an ‘insulinoma’. These tumours cause signs such as collapse at exercise and very low blood sugar. Insulinomas are difficult to cure because they spread to other tissues very commonly. The diagnosis of an insulinoma requires the demonstration of the simultaneous occurrence of low blood glucose levels and either normal or elevated blood insulin levels. The diagnostic imaging of insulinomas in dogs is preferably conducted by taking computed tomography scans. In general, the surgical treatment of insulinomas is the preferred therapy because it results in the best survival times, but many dogs also require medical treatment at some point. The prognosis of dogs with insulinomas is guarded, because clinical symptoms relating to low blood sugar almost always return due to tumour regrowth or spread. This review paper aims to provide a summary and update the current recommendations in the veterinary literature for the diagnosis, treatment and clinical outcome of insulinomas in dogs. The contents of this review are comprehensive and relevant to veterinarians that work at all levels and will inform and advance our understanding of insulinomas in dogs.

**Abstract:**

The most common pancreatic tumour in dogs is the insulinoma. Canine insulinomas are considered to be malignant in more than 95% of the cases because they almost always tend to metastasise. The diagnosis of an insulinoma requires the demonstration of the simultaneous occurrence of hypoglycaemia and blood insulin levels that are within or above the high end of the reference interval. The staging of canine insulinomas is preferably conducted by contrast-enhanced computed tomography. In general, surgical treatment is the most effective because of it results in long survival times, but many dogs also require medical treatment at some point. The prognosis of canine insulinomas is guarded as clinical hypoglycaemia almost always returns due to metastasis or tumour regrowth. This review aims to provide a summary and update the current recommendations in the veterinary literature for the diagnosis, treatment and prognostication of canine insulinomas.

## 1. Introduction

The most common pancreatic tumour in dogs is the insulinoma [1]. Dogs of middle-sized to large breeds are most commonly diagnosed with insulinomas. There is no sex predisposition and no clear breed predisposition for insulinomas, but there appear to be more cases in German Shepherds, Irish Setters, Boxers, Golden Retrievers, Poodles, Fox Terriers, Collies and Labrador Retrievers, although smaller breeds like West Highland White Terriers have also been reported to develop them [2,3,4]. Based on data from 214 dogs from eight publications, dogs with insulinomas are on average 9.1 years old (range, 3–15 years) when they are diagnosed with the disease [1,2,5,6,7,8,9,10].

Besides insulin, neoplastic b-cells in dogs produce glucagon, somatostatin, gastrin, serotonin, growth hormones and pancreatic polypeptide, according to immunohistochemistry studies [11,12,13]. Insulinomas in dogs also contain amphicrine cells expressing both insulin and pancreatic lipase [14]. In humans, around 50% of endocrine pancreatic neoplasms have been shown to produce multiple hormones in immunocytochemistry tests, but the occurrence of mixed clinical syndromes is very rare [15]. Mixed clinical syndromes have not been described in dogs with endocrine pancreatic tumours, hence, insulinomas are named after insulin, its principal hormone, and its most common clinical sign: hyperinsulinaemia-induced hypoglycaemia.

In most cases, primary canine insulinomas are nodular tumours with a diameter of less than 2.5 cm [2,10]. Most insulinomas occur in the left or right pancreatic limb, rather than in the pancreatic corpus [1,2,7,16]. The presence of multiple primary tumours has been reported in 4–14% of cases [2,10]. Despite the lack of histological criteria for malignancy, insulinomas almost always metastasise so they are regarded as malignant in more than 95% of cases [3,4]. In 40 to 50% of the dogs, insulinoma metastases are already visible at surgery, primarily in the abdominal lymph nodes and/or liver [2,3,7].

Usually, canine insulinomas are diagnosed by biochemical testing following a high level of clinical suspicion. The staging of it is performed according to the World Health Organization’s TNM (tumour, node, metastasis) system (Table 1) [17]. There are three stages of insulinomas in dogs: T1N0M0 (clinical stage I), T1N1M0 (clinical stage II), and T0N0M1, T1N0M1 or T1N1M1 (clinical stage III). Surgery is usually the best treatment option in most cases.

A decade has passed since a comprehensive review on canine insulinomas has been published [18]. Since then, 35 original research papers have been published investigating canine insulinomas, and the clinical management of dogs with insulinomas has changed significantly. Hence, the objective of this paper is to review the current recommendations in the veterinary literature for the diagnosis, treatment and prognostication of canine insulinomas.

## 2. Pathophysiology

The hypersecretion of insulin by insulinomas increases the blood insulin levels. By inhibiting glycogenolysis and gluconeogenesis, elevated insulin levels suppress the hepatocyte glucose secretion. Furthermore, high insulin concentrations trigger muscle and fat tissues to absorb glucose. The excretion of insulin is tightly controlled in normal β-cells by the blood glucose levels. Glucose enters β-cells via the facilitative glucose transporters (GLUTs), which is an insulin independent process in pancreatic β-cells, which is contrary to most other cells. As the blood glucose concentrations rise, the insulin secretion gradually increases, and eventually plateaus. On the other hand, insulin secretion is inhibited when the blood glucose concentration decreases below that of a physiologic set point [19]. The insulinomas secrete excessive insulin because the negative feedback mechanism that is initiated by falling blood glucose levels is disrupted in the neoplastic β-cells. Therefore, the insulinomas cause profound hypoglycaemia [20]. The precise mechanisms underlying the excess amount of insulin secretion by the insulinomas and the impaired cellular response to hyopglycaemia are not well understood. A recent study, however, demonstrated an apparent, upregulated expression of glucokinase (GCK) in three canine insulinomas which were compared to paired normal pancreatic tissues. GCK catalyses the first step in the cellular glucose metabolism and acts as a glucose sensor in the cells where it is expressed. It has been suggested that the overexpression of GCK in canine insulinoma cells may partly explain why insulinomas secrete excessive amounts of insulin [21].

The stimulation of the autonomic nervous system and the absence of an energy substrate to the central nervous system cause clinical signs of hyperinsulinaemia-induced hypoglycaemia. Neurologic signs occur because nervous tissue can use only glucose as its energy source and the diffusion of glucose across the blood–brain barrier and cerebral oxidation are severely impaired when the blood glucose levels are low [22].

## 3. Clinical Features

The most common clinical symptoms of insulinomas in dogs are the symptoms of neuroglycopaenia, namely: seizures, generalised weakness, posterior paresis and collapse (Table 2) [23,24]. Dogs may also demonstrate muscle tremors, nervousness and hunger due to the hypoglycaemia-induced activation of the autonomic nervous system. The clinical symptoms of canine insulinomas commonly appear intermittently in situations leading to increased glucose utilisation. As a result, hypoglycaemic episodes are preceded by fasting, exercise, excitement or stress in the early stages [19]. Glucose nadir, the rate of the decrease in the blood glucose concentration and hypoglycaemia duration all effect the severity of the clinical symptoms [25]. Compared to a blood glucose concentration of 2 mmol/L that develops rapidly over a few hours, a gradual decline to 2 mmol/L (normal reference: 4.2–5.8 mmol/L) over an extended period is less likely to indicate the clinical signs of hypoglycaemia. The clinical signs are usually absent between the hypoglycaemic episodes in the affected dogs. If the hypoglycaemia is prolonged and severe, cerebral cortical laminar necrosis may eventually develop, resulting in coma and death [26].

## 4. Clinical Pathology

In dogs with insulinomas, routine complete blood counts, serum biochemistry, and urinalysis are mostly used to detect the symptoms that are within the normal reference range, with the exception of the presence of hypoglycaemia [19]. Although these routine laboratory investigations are not diagnostic for insulinoma, the results of these tests are helpful for ruling out less likely other disorders that can cause hypoglycaemia. Because blood glucose concentrations fluctuate significantly throughout the day, it is possible to find a blood glucose concentration that is within the reference range [27]. It may be necessary to take fasting samples and repeat testing to confirm hypoglycaemia in these cases.

There have also been reports of mildly increased plasma levels of albumin, alkaline phosphatase, alanine transaminase, bile acids, amylase and lipase in dogs with insulinomas, and mildly decreased urea, creatinine and globulin concentrations. These abnormalities, however, are not specific and have no diagnostic value [1,5].

## 5. Biochemical Diagnosis

Historically, the presumptive diagnoses of canine insulinomas have been based on signalment and clinical history, combined with the Whipple’s triad [28]. The triad consists of demonstrating (1) the presence of hypoglycaemia, (2) the presence of the clinical symptoms that are associated with a subnormal blood glucose concentrations, and (3) the relief of the clinical symptoms after glucose administration or feeding. If there is a clinical suspicion of an insulinoma, but a dog is not hypoglycaemic on presentation (e.g., due to increased catecholamines during hospitalisation), it may be required that the clinician fast the dog to demonstrate hypoglycaemia. Fasting should be carefully managed by an hourly evaluation of the blood glucose concentration because blood glucose levels decrease before hypoglycaemic signs occur possibly causing rapid and serious symptoms in dogs with insulinoma [5,29]. Most dogs with insulinomas that are fasted will demonstrate hypoglycaemia within 24 h [3].

The Whipple’s triad fits all of the causes of hypoglycaemia and therefore, the next step in the diagnostic process is to rule out differential diagnoses. In the elderly dog, aside from insulinoma, obtaining common differential diagnoses of hypoglycaemia includes performing spurious laboratory results, hypoadrenocorticism, hepatic insufficiency, portosystemic shunts, sepsis, and nonpancreatic neoplasia producing incompletely processed insulin-like growth factors (e.g., hepatocellular carcinoma, leiomyosarcoma, and metastatic mammary carcinoma and lymphoma) [4,30,31]. Less common differential diagnoses are juvenile hypoglycaemia, hunting dog hypoglycaemia, glycogen storage disease, glucagon deficiency and nesidioblastosis. Finally, the iatrogenic causes of hypoglycaemia include the administration of drugs like insulin and sulfonylurea [5,29,32]. Differential diagnoses of canine insulinoma can be excluded by simultaneously measuring their insulin levels at the time when the hypoglycaemia is diagnosed. Historically, several other diagnostic tests have been used in the diagnostic work-ups of canine insulinoma patients, including the amended insulin-to-glucose ratio, the intravenous glucose tolerance test and the glucagon tolerance test. These tests are now considered obsolete as they lack specificity and could be risky because they can trigger hypoglycaemic episodes [3,19,29].

In canine insulinomas, circulating insulin concentrations are generally within or above the reference interval (2–21 μU/mL) [5]. The concurrent occurrence of blood glucose that is below 3.5 mmol/L and plasma insulin that is above 10 μU/mL is characteristic of a canine insulinoma [25]. Although plasma insulin concentrations that are above the high end of the reference range are most commonly seen in 56–83% of dogs with insulinomas, plasma insulin can also be within the reference range [5,6]. The plasma insulin concentration should, however, be reduced to below its reference range when the blood glucose is lower than 3.5 mmol/L, and the absence of this response marks the occurrence of inappropriate insulin secretions [25].

## 6. Tumour Localisation

Once the diagnosis of the insulinoma is confirmed, every effort should be made to determine the anatomical localisation of it within the pancreas. It is important to know the anatomical localisation of the insulinomas prior to surgery since it facilitates the choice between open and laparoscopic pancreatic surgery and between the enucleation or resection by a partial pancreatectomy.

The techniques that are most commonly used to demonstrate tumours in the pancreas include transabdominal ultrasonography and computed tomography (CT). Although ultrasonography is widely available and therefore, commonly used in general practice, it has a low sensitivity of 36% in detecting canine insulinoma [33]. Additionally, ultrasonography alone is insufficient for staging of canine insulinomas, as highlighted by the Robben et al. (2005) study in which none of the five lymph node metastases were detected by ultrasonography [33]. For the achievement of detailed preoperative TNM staging, it is therefore crucial to refer patients to institutions that have a CT scanners that are available. The TNM stage is an important prognostic factor of canine insulinomas [4]. Knowing their dog’s insulinoma TNM stage, will allow clients to make a fully informed decision regarding the treatment options, hence, a CT scan should be an essential part of the diagnostic staging of an insulinoma.

Conventional pre- and post-contrast CTs have been shown to have a sensitivity of 71% and 40% for the detection of primary insulinomas and lymph node metastases, respectively [33]. More recently, dual- and triple-phase contrast-enhanced CT (CECT) techniques have been developed, and the CT images of canine insulinomas have been obtained during the arterial, portal and delayed venous phases after the administration of an intravenous contrast agent [34,35]. CECT had a high sensitivity (96%) in detecting primary insulinomas in a case series of 27 dogs with insulinomas [16]. The sensitivity of CECT scans to detect lymph node metastases was 67% and the sensitivity to detect liver metastases was 75%. While there was no specific post-contrast phase during which the insulinomas were best visualised, major location errors were more likely to occur in single- or double-phase CECT scans than they were in triple-phase CECT scans. According to another study of 35 dogs, insulinomas tend to be hyperattenuated during the arterial phase, and hypoattenuation or isoattenuation are much less common [36].

Although magnetic resonance imaging (MRI) has not been frequently used to image the pancreases of dogs, a small case series including four dogs investigated its potential for detecting canine insulinomas [37]. All four of the insulinomas displayed a high-intensity signal on the T2-weighted fat-saturated images, whereas the tumours were primarily isointense-to-normal on the post-contrast T2-weighted fat-saturated images.

Most recently, a pilot study investigated the utility of fluorine-18 fluorodeoxyglucose positron emission tomography-computed tomography (^18^F-FDG PET-CT) for the identification of primary and metastatic canine insulinomas [35]. The technique of ^18^F-FDG PET-CT uses the glucose analogue ^18^F-FDG to assess the tumour’s metabolic activity. This imaging modality has proven to be effective for the imaging of human malignant insulinomas with 88.9% of human patients having avid lesions on the ^18^F-FDG PET-CT images. The three canine insulinomas that were included in the Walczak et al. (2022) study, however, were inconsistently avid on the ^18^F-FDG PET-CT images, and it was concluded that the unpredictable tumoural avidity limits the value of ^18^F-FDG PET-CT for staging canine insulinomas [38].

In spite of new developments in preoperative diagnostic imaging methods, exploratory laparotomy remains the most dependable technique to detect primary and metastatic insulinomas. The meticulous intraoperative visual and tactile investigation of the pancreas and the nearby structures, with or without the administration of intravenous methylene blue (3 mg/kg, given over 30–40 min), exposes the most primary insulinomas and metastatic lesions. The use of intravenous methylene blue, however, is not routinely recommended since it can cause fatal haemolytic anaemia or acute renal failure [19]. When an intraoperative examination fails to reveal the tumour, an intraoperative ultrasound can be used to visualise both the primary insulinoma and liver metastases that may have occurred [33].

## 7. Treatment

In general, surgical treatment is the most effective because of it results in long survival times (STs), but many dogs also require medical treatment at some point [1,4,39].

### 7.1. Medical Therapy

#### 7.1.1. Emergency Treatment

An acute hypoglycaemic crisis resulting in sudden and serious seizures in dogs should be treated immediately because the shorter the duration of the hypoglycaemia, then the lower the risk of irreversible brain damage is [40]. Initially, 1 mL/kg of 20% glucose should be administered slowly and intravenously over 5–10 min [18,41]. If this stabilizes the patient, a small meal should be fed to them, and long-term medical treatment should be initiated. In the case of intractable seizures, a continuous rate infusion of 2.5–5% glucose should be started at 3–4 mL/kg/h. As a next step, dexamethasone 0.5–1 mg/kg can be added to the intravenous fluids and administered over 6 h, which can be repeated if necessary every 12–24 h [18,41]. Additionally, a glucagon bolus of 50 ng/kg can be given, which is followed by a continuous rate infusion that starts at 5–10 ng/kg/min [18,41,42,43]. If the dog is still seizuring after the normalisation of the blood glucose levels, diazepam (1 mg/kg) or propofol (2–6 mg/kg) can be administered. Ultimately, if everything else fails, the dog can be anaesthetised with pentobarbital for 4–8 h while continuing one is the treatment outlined above and the urgent surgical resection of the insulinoma should be considered [41].

#### 7.1.2. Long-Term Management

Dogs with an insulinoma should be fed four to six small meals a day and their diet should contain high levels of proteins, fats and complex carbohydrates. This type of diet that is without simple carbohydrates, decreases the postprandial hyperglycaemia and thus, prevents a pronounced stimulus for tumour insulin release [18]. Exercise needs to be restricted to short leash walks only which may also help to prevent clinical hypoglycaemia [26]. The use of additional medications should be initiated if the clinical signs persist despite frequent feedings and restraints from physical activity.

Diazoxide is the preferred drug to treat insulinoma-induced hypoglycaemia. Diazoxide blocks pancreatic insulin release, stimulates hepatic gluconeogenesis and glycogenolysis and inhibits glucose uptake by tissues [1,3,4]. The recommended starting dose of diazoxide is 5 mg/kg *per os* twice daily, which can be progressively increased if it is needed to 30 mg/kg twice daily [19]. Side effects are rare, but anorexia, vomiting and ptyalism have been reported to have occurred. These side effects may be prevented by administering diazoxide with meals. The contraindications for the use of diazoxide in dogs are liver, kidney or heart failure [19,44].

If diazoxide is not available at the pharmacy, or if long-term diazoxide treatment is too expensive, then glucocorticoids can be used as an alternative. Glucocorticoids, such as prednisolone, increase the rate of hepatic gluconeogenesis and glycogenolysis and antagonise the effects of insulin at the cellular level [41]. The recommended starting dose of prednisolone is 0.25 mg/kg twice daily *per os*. In the refractory cases, prednisolone doses of up to 2.0–3.0 mg/kg twice daily can be used, although dosages that are higher than 1.1 mg/kg twice daily are considered to suppress the immune system [19]. Additionally, high glucocorticoid dosages often give rise to an iatrogenic Cushing syndrome [44].

On top of the frequently used drugs that are outlined above, the use of somatostatin analog octreotide and nitrosourea compound streptozocin have been described for the treatment of canine insulinomas [8,13,45,46]. These therapies, however, have not become well established in the treatment of canine insulinomas and can be considered obsolete due to the questionable efficacy of them and a high risk of severe adverse effects occurring [8,46,47,48].

Finally, most recently, a case report and two small retrospective case series described the use of tyrosine kinase inhibitor toceranib phosphate for canine insulinomas [49,50,51]. Although some dogs in these studies were reported to have long-term glycaemic control following toceranib phosphate therapy, it is currently unclear whether this effect could be attributed to toceranib phosphate. The major limitations of these studies were that they had a retrospective nature, and there was heterogeneity due to them using small study groups. Hence, prospective clinical trials would be warranted to examine the efficacy of toceranib phosphate in the treatment of canine insulinomas.

### 7.2. Surgical Therapy

#### 7.2.1. Preoperative Considerations and Anaesthesia

The following protocol is favoured by the author to prepare dogs with a suspected insulinoma for anaesthesia and surgery. For the prevention of fasting hypoglycaemia, dry food should be withheld for 12 h before surgery, but canned food can be fed to them for up to 6 h beforehand. Dogs that are suffering from clinical hypoglycaemia should be given easily digestible liquid food preparations for up to 1–2 h before surgery. In the immediate preoperative period, 1–5 mL of 50% dextrose should be administered to them intravenously slowly over 10 min if the clinical signs occur. An intravenous infusion of a balanced electrolyte solution containing 2.5–5.0% dextrose should be administered continuously to the dogs while they are under anaesthesia, and their blood glucose levels should be closely monitored. The rate of intravenous dextrose infusion may need to be adjusted if the tumour is manipulated during the surgery, leading to increased insulin secretion, which can lead to rebound hypoglycaemia. Once the primary tumour is removed, the dextrose infusion is typically stopped, since normoglycaemia is usually restored within minutes, or hyperglycaemia is induced [25].

#### 7.2.2. Surgical Techniques

Regardless of the preoperative imaging results, during the surgery, the entire pancreas should be assessed to check for the location of the insulinoma. Depending on its location in the pancreas, an insulinoma can be excised by a local enucleation if the insulinoma is located in or close to the pancreatic corpus, or by partial pancreatectomy if it is located in the right or left pancreatic limb. During the local enucleation of insulinomas in the pancreatic corpus, utmost care should be taken to avoid the disruption of the pancreatic ducts and the pancreaticoduodenal arteries [19].

A partial pancreatectomy is frequently done using either the suture–fracture method or bipolar vessel sealing [52]. Using the suture-fracture technique, after incising the mesoduodenum or omentum bilaterally to the pancreas, the sutures are used to encircle the pancreas, and they are placed immediately proximal to the insulinoma. Upon the tightening of the ligatures, they crush through the parenchyma of the pancreas, and result in the presence of ligate vessels and ducts. The part of the pancreas that is distal to the ligatures, including the insulinoma, is excised. When one is using a bipolar vessel sealant device for a partial pancreatectomy, there is no need for sutures to be on the pancreas because the sealant device will provide secure and rapid haemostasis, thereby sealing tissue bundles and blood vessels up to 7 mm in diameter [53]. Bipolar vessel sealing is the preferred technique for a partial pancreatectomy, especially since it improves the surgical performance in lesions that clinicians have difficulty accessing coin comparison to the suture-fracture technique [52].

The most recent improvement of the surgical technique for the resection of canine insulinomas has been the use of laparoscopy [54,55]. A laparoscopic partial pancreatectomy can be applied to dogs with insulinomas in the distal two-thirds of the right or left limb of the pancreas. Based on the location of the insulinoma, either a ventral or a flank-approach can be used. Selected abdominal lymph node metastases can be accessible for their laparoscopic resection as well, however, laparoscopy is contraindicated in those cases in which insulinomas have extensive lymph nodes or liver metastases.

An assessment of the metastatic disease is made by the gross inspection of the lymph nodes in the abdomen and the liver, as well as by measuring the blood glucose levels after the dextrose infusion has been stopped. The excision of any lymph node that appear to be macroscopically enlarged is recommended. For liver metastases, it is necessary to debulk the metastases to increase the effectiveness of the medical adjuvant therapy.

#### 7.2.3. Postoperative Complications

Acute pancreatitis is a major concern after pancreatic surgery and its occurrence has been demonstrated in 10% of dogs after an insulinoma resection has been performed [7,56]. Although only 1:10 dogs will experience a complete form of pancreatitis, inappetence and vomiting postoperatively occurred in 27.3% and 24.2% of 33 dogs that underwent surgery for the removal of the insulinomas [57]. Persistent hypoglycaemia has been reported in up to 23% of dogs after they underwent surgery and it warrants a guarded prognosis as it usually indicates that residual insulinoma cells were left behind in the patient [56,58]. Hyperglycaemia can also develop post-operatively, but it is generally transient until the remaining b-cells recover their function. Dogs can, however, develop persistent diabetes mellitus, especially when the bulk of the pancreas has been resected. Del Busto et al. (2020) reported that postoperative hyperglycaemia was identified in 33% of dogs after the resection of their insulinomas, of which, 19% developed persistent diabetes mellitus [56].

## 8. Prognosis

Insulinomas in dogs have a reserved prognosis and clinical hypoglycaemia almost always returns for them, due to metastasis or tumour regrowth occurring. Combining medical therapy and surgery in dogs results in a significantly better prognosis than medical treatment alone does [39]. The median disease-free (DFI) interval and median ST of the dogs after they underwent a partial pancreatectomy were 12 months (0–55 months) and 14 months (0–51 months), respectively [1,3,7,23,39,56]. However, the median ST of 21 dogs that were treated medically was just 4 months (0–18 months) [1,24,39]. The TNM stage that they were in, age, and their postoperative blood glucose levels were also prognostic factors. Caywood et al. (1988) found that dogs with a clinical stage I insulinoma had a median DFI of 14 months, which was significantly longer than the median DFI of 1 month of dogs with clinical stage II and III insulinomas [4]. A median ST of 18 months was observed in the dogs with clinical stage I and II insulinomas as compared to that of 6 months in the dogs with a clinical stage III insulinoma [4]. Contrary to the latter study, Buishand et al. (2010) did not find a significant difference between the dogs with insulinomas that were confined to the pancreas and dogs with metastatic insulinomas in terms of their DFIs [10]. Rather, clinical stage III insulinomas were found to remain subclinical for significantly longer in the dogs after they underwent surgery than the clinical stage I and II insulinomas did. In addition, the Ki67 index was found to be a biomarker of a canine insulinoma that can be used to predict the clinical outcome [10]. Dogs with insulinomas with a Ki67 index > 2.5% had significantly shorter DFIs and STs when they were compared to dogs with insulinomas with a Ki67 index ≤ 2.5%. The prognosis of young dogs has been reported to be worse than that of older dogs [4]. More recent studies, however, have reported that age is not a significant prognostic factor [7,10,58]. The median ST of dogs that are hyperglycaemic, or normoglycaemic when they are in the postoperative phase, is significantly longer than that of dogs with hypoglycaemia after surgery (680 and 90 days, respectively) [7]. After surgically treating the insulinomas, 80 percent of them achieved a hypoglycaemia resolution in the immediate postoperative period, whereas 20% of them remained persistently hypoglycaemic. A median ST of 561 days was recorded for all of these dogs. The median ST for the dogs with hypoglyaemia that resolved was 746 days. After 2 years of being postoperative, 44% of patients who had hypoglycaemia resolved the recurrence of this. Cleland et al. (2021) found that the pathological stage predicted the occurrence of persistent hypoglycaemia postoperatively, which, in turn predicted the ST [58].

## 9. Future Directions

Despite current multimodal treatment protocols that are being used, as are outlined above, the prognosis for canine insulinomas remains guarded in most cases because of tumour regrowth and/or the presence of micrometastases during surgery. Human malignant insulinomas, that have spread beyond the pancreas have an unfavourable prognosis as well because of the low success rate of the current therapies [59]. Hence, the development of new targeted therapies is warranted to improve the prognosis of dogs and humans with insulinomas.

Comparative oncology focuses on the use of spontaneous tumours in pets as sophisticated models for the investigation of human cancer biology and therapy. Numerous studies have been conducted since the publication of the canine genome in 2005 demonstrating that cancer in dogs is an excellent model for human cancer [60,61]. In dogs and humans, naturally-occurring cancers are characterised by a similar histology, tumour biology, progression of disease, and response to conventional therapy [62]. Like dogs with insulinoma, humans with functional insulinomas demonstrate clinical signs of hypoglycaemia secondary to insulin hypersecretion, and surgical and medical treatment strategies are the same in both species. Moreover, in 90% of canine and human cases, primary insulinoma are solitary tumours, and histologically, insulinomas share the same features in dogs and humans [1,39,63,64]. Finally, canine insulinomas have been shown to share molecular similarities with human insulinomas as well, further supporting the value of canine insulinoma as an interesting study model for human insulinoma [65].

Veterinary comparative oncology studies from the past decade have improved our understanding about the driving factors and critical survival genes in insulinomas. The most important findings from these studies are:

- Insulinoma cancer stem cells can be identified using the cell surface marker CD90, which is also known as Thy-1, which is a potential therapeutic target. A zebrafish embryo insulinoma xenograft model was treated with anti-CD90 monoclonal antibodies, which resulted in the decreased viability of the injected insulinoma cells. Immunohistochemistry tests confirmed the expression of CD90 in clinical insulinoma samples, with there being positive staining in insulinoma cells, intra-tumoural fibroblasts and vascular endothelium. Consequently, anti-CD90 monoclonals are a promising new adjuvant therapy modality that either targets insulinoma cells directly or targets the insulinoma microenvironment [66].
- Another subpopulation of canine and human insulinoma cancer stem cells, which is characterised by the expression of *OCT4, SOX2, SOX9, CD133* and *CD34*, exhibited the overexpression of Notch-signalling related genes. Cancer stem cells were more sensitive to 5-fluorouracil (5-FU) in vitro, and in an in vivo chicken chorioallantoic membrane tumour model, when the Notch pathway was inhibited with a gamma secretase inhibitor. By inhibiting Notch-signalling and targeting 5-FU synergistically, these findings demonstrated the synergetic attenuation of enriched INS cancer stem cell populations, which may be exploitable as a therapy in the future [67].
- An RNA sequencing study of canine insulinomas showed that early clinical stage insulinomas and the normal pancreas had similar genetic profiles, while late clinical stage insulinomas genetically resembled metastatic lymph nodes. Therefore, it appears that that markers of malignant behaviour can be found at the primary site of the disease [68].

Although additional studies are required, ultimately, these fundamental findings have the potential to be translated to new, more precise insulinoma treatments.

## Figures and Tables

**Table 1:** TNM classification of canine insulinoma.

**Primary Tumour (T)**
T0—no evidence of primary insulinoma
T1—insulinoma present in pancreas
**Abdominal Lymph Nodes (N)**
N0—negative nodes
N1—positive nodes
**Distant Metastasis (M)**
M0—no distant metastasis
M1—distant metastasis

**Table 2:** Most common clinical signs in 320 dogs with insulinomas [23,24].

Clinical Signs	Number of Dogs (Percentage)
Seizures	167 (52%)
Weakness	134 (42%)
Posterior paresis	106 (33%)
Collapse	89 (28%)
Muscle fasciculations	60 (19%)
Ataxia	59 (18%)
Polyphagia	22 (7%)
Polyuria and polydipsia	18 (6%)

## Data Availability

Not applicable.
